# Urban wastewater bacterial communities assemble into seasonal steady states

**DOI:** 10.1186/s40168-021-01038-5

**Published:** 2021-05-20

**Authors:** Emily Lou LaMartina, Aurash A. Mohaimani, Ryan J. Newton

**Affiliations:** 1 School of Freshwater Sciences, University of Wisconsin-Milwaukee, Milwaukee, WI 53204 USA; 2 Present Address: Analytical Technologies, Biogen, 5000 Davis Dr, Morrisville, NC USA

**Keywords:** Wastewater, Sewer, Microbial ecology, Urban microbiome, Time series, Seasonal dynamics

## Abstract

**Background:**

Microorganisms in urban sanitary sewers exhibit community properties that suggest sewers are a novel ecosystem. Sewer microorganisms present both an opportunity as a control point for wastewater treatment and a risk to human health. If treatment processes are to be improved and health risks quantified, then it is necessary to understand microbial distributions and dynamics within this community. Here, we use 16S rRNA gene sequencing to characterize raw influent wastewater bacterial communities in a 5-year time series from two wastewater treatment plants in Milwaukee, WI; influent wastewater from 77 treatment plants across the USA; and wastewater in 12 Milwaukee residential sewers.

**Results:**

In Milwaukee, we find that in transit from residences to treatment plants, the human bacterial component of wastewater decreases in proportion and exhibits stochastic temporal variation. In contrast, the resident sewer community increases in abundance during transit and cycles seasonally according to changes in wastewater temperature. The result is a bacterial community that assembles into two distinct community states each year according to the extremes in wastewater temperature. Wastewater bacterial communities from other northern US cities follow temporal trends that mirror those in Milwaukee, but southern US cities have distinct community compositions and differ in their seasonal patterns.

**Conclusions:**

Our findings provide evidence that environmental conditions associated with seasonal change and climatic differences related to geography predictably structure the bacterial communities residing in below-ground sewer pipes.

Video abstract

**Supplementary Information:**

The online version contains supplementary material available at 10.1186/s40168-021-01038-5.

## Background

Urban sewers collect wastewater from a variety of sources, including stormwater, industrial waste, and residential sewage. Sewer pipes transport wastewater to wastewater treatment plants (WWTPs), where nutrients and microorganisms are removed and select microorganisms are cultivated to aid treatment processes [1]. Imbalanced WWTP microbial communities can disrupt treatment and create challenging and costly problems. For instance, WWTPs typically settle activated sludge to separate it from treated wastewater, but overgrowth of filamentous bacteria causes poor settling, which deteriorates effluent quality and may require significant process alterations to remedy [2]. The goal of wastewater treatment is to foster beneficial microbial communities and remove problematic ones, and WWTP influent can be a source of each [3,5].

Sewers serve as more than conveyance for wastewater. The consistency in sanitary sewer microbial community composition suggests that sewers represent a recently formed ecosystem [6]. Some resident sewer microbes induce pipe corrosion [7, 8], display pathogenic lifestyles [9], or propagate antibiotic resistance genes [10, 11], including those that survive treatment and persist in receiving waters [12,15]. Aging and inadequate infrastructure also introduces sewer bacteria to the environment by leaching wastewater through corroded pipes [16,18] or through deliberate release during sewer overflows [19, 20]. Sewage discharge regularly impairs recreational waters, causes coastal beach closures, and poses a significant risk to human health [21]. Despite the potential importance of resident sewer bacteria, there is not a thorough understanding of whether the majority of these microorganisms exhibit predictable abundance patterns through time or among sewer systems, partition to various substrates in wastewater, or survive for prolonged periods in natural aquatic systems after discharge.

Many aquatic ecosystems undergo seasonal changes that drive biological change, which in turn creates repeating and predictable microbial community structures and ecosystem services [22,25]. As sewers are a primarily aquatic environment, it is possible the resident microbial communities also exhibit temporal community assembly patterns. Initial studies suggest this may be the case. Guo et al. [26] revealed diurnal trends in WWTP influent microbial communities that were driven by change in flow rate between day and night, where low flow resulted in less sloughing of pipe bacteria and thus a change in composition. Although this study provided evidence of repeatable microbial dynamics, these dynamics were driven by short-term physical factors. To the best of our knowledge, no study has analyzed whether pre-treatment wastewater microbial communities are also impacted by longer-term changes (months or years) to their environment. Uncovering patterns of assembly by sewer microbial communities will aid in designing models to predict wastewater composition, enable targeted treatments for microorganisms of interest, and identify whether temporal community variation relates to altered human and/or environmental health risks from untreated discharge.

To address this knowledge gap, we used 16S rRNA gene sequencing to analyze bacterial communities in three wastewater datasets: (1) a 5-year time series of WWTP influent sampled once per month from two facilities in Milwaukee, WI, USA; (2) WWTP influent from 77 facilities in the USA sampled during three seasons in a single year; and (3) wastewater from 12 sewers in four distinct residential Milwaukee neighborhoods. To assess the mixing of microbiomes, or the community within a community, we identified the human-associated bacterial assemblage in wastewater and analyzed it independently from the rest of the bacterial community. We hypothesize that (1) the majority of sewer pipe bacteria are not from human waste and persist year-round; (2) wastewater resident bacterial communities follow predictable, seasonal patterns in assembly; and (3) temporal community assembly trends in Milwaukee will be similar to wastewater from other northern US cities.

## Methods

### Sample collection

#### Milwaukee time series

We collected 24-h flow-proportional composite samples of WWTP influent once a month for 5 years from Jones Island (JI) and South Shore (SS) water reclamation facilities in Milwaukee, WI, USA. At JI, 100 mL aliquots from continuous water sampling at three sample points were combined into a final composite sample. Each sampling point has variable sampling frequencies depending on flow at that location. Under low flow (range = 10 million gallons per day (MGD) to 120 MGD depending on sample point), the volumes that trigger an aliquot collection are (1) 0.2 MG, (2) 0.5 MG, and (3) 0.6 MG, respectively, while under high flow (range = > 60 MGD to > 120 MGD) the volumes that trigger an aliquot collection are (1) 0.8 MG, (2) 1.0 MG, and (3) 1.4 MG. At SS, a single composite sample was collected. Under low flow conditions (< 100 MGD) a 100-mL aliquot is collected at 0.71.9 MG, while under high flow conditions ( 100 MGD) an aliquot is collected at 1.94.0 MG. JI influent samples spanned each month from January 2013 to February 2018, except 2 months (November 2014 and March 2015; *n* = 60). SS influent samples spanned October 2014 to December 2017, except 5 months (November 2014, March 2015, May 2015, November 2015, and June 2017; *n* = 34). After collection, we filtered 10-mL onto 0.22-m mixed cellulose ester filters (47-mm diameter, Millipore Sigma) and stored at 80 C for up to 5 years before extracting DNA. The Milwaukee Metropolitan Sewerage District measured environmental parameters in each sample (File S1).

#### Across USA

As described previously in Newton et al. 2015 [27], sewage influent samples (*n* = 204) were collected from 77 wastewater treatment plants (WWTPs) in 72 US cities around August 2012, January 2013, and May 2013 (Figure S1 and Table S1). Wastewater samples included a variety of collection setups, ranging from single time-point grab samples to 24-h flow weighted composites. All samples were collected, stored in a refrigerator on site for < 24 h and shipped overnight to our lab for immediate filtering onto 0.22-m mixed cellulose ester filters. For specific sample collection details, see Newton et al. 2015 [27].

#### Milwaukee neighborhood sewer samples

We collected 5-h time-paced composite (04000900 h, with 50 mL aliquots taken every 15 min) samples from three sewers in each of four neighborhoods, Elm Grove, South Milwaukee, North Milwaukee, and New Berlin in the Milwaukee sewerage district on the 15th and 17th of December 2015 (*n* = 24; Figure and Table S2). Each residential sewer sample represented a 200600 household drainage area. From these samples, we filtered 25-ml onto 0.22-m mixed cellulose ester filters (Sigma Millipore) and stored them at 80 C for up to 3 months before extracting DNA.

### DNA extraction

We crushed frozen filters in their storage tubes using a sterile metal spatula, added a bead-beating matrix and buffers from the FastDNA Spin Kit for Soil (MP Biomedicals), and bead beat for 1 min. We then extracted DNA following the FastDNA Spin Kit for Soil protocol.

### PCR and amplicon sequencing

#### Milwaukee time series

We amplified the V4V5 region of bacterial 16S rRNA genes in wastewater samples using primers 518F and 926R [28]. The following setup was used: 12.5-l 2 KAPA HiFi HotStart ReadyMix PCR (Roche), 1.5-l of each 5-M forward and reverse primer working solutions, 7.5-l sterile water, and 2-l 100-diluted DNA template. PCR was run on a vapo-protect Mastercycler pro S (Eppendorf) under the following conditions: 95 C for 5 min; 22 cycles of 98 C for 20 s, 55 C for 15 s, 72 C for 1 min; 72 C for 1 min; 4 C hold. We included one negative control and one mock community (#HM-782D, BEI Resources). Triplicate PCRs were pooled and cleaned with Agencourt AMPure XP beads (Beckman Coulter), following the manufacturers protocol. Sample libraries were prepared according to the Illumina MiSeq protocol in the Nextera XT Index kit (Illumina). Indexed PCR amplicons were cleaned with AMPure beads and normalized with the SequalPrep Kit (ThermoFisher Scientific). Sequencing was carried out on an Illumina MiSeq with 2 250 chemistry at the Great Lakes Genomics Center (greatlakesgenomics.uwm.edu).

#### Across USA and Milwaukee residential sewers

All procedures were the same as described above, except that sample library preparation and sequencing was carried out at the Marine Biological Laboratory (MBL).

### Sequence processing

Forward and reverse reads were quality-filtered using FastQC [29] and primers were trimmed with Cutadapt [30]. We processed the three wastewater datasets simultaneously with the R package DADA2 [31], following the protocol at http://benjjneb.github.io/dada2/tutorial.html, with the following exceptions: during filtering, reads were truncated at 230 bp, and reads with quality scores lower than 10 were removed; after merging, sequences were removed that did not have lengths within 5% (355 to 393) of the median sequence length (374 bp). Taxonomy was assigned to resulting amplicon sequence variations (ASVs) using Silva v. 132 [32]. ASVs that were not classified as bacteria or were classified as mitochondria or chloroplasts were removed. Contaminant ASVs from the mock community and negative control were identified with the R package Decontam [33] and subsequently removed.

### Primer design, ddPCR, and qPCR

We designed primers to target unique 16S rRNA V4-V5 gene regions belonging to one *Cloacibacterium* and one *Flavobacterium* ASV (Table S3). Non-target ASVs of the same genus were included as negative controls for primer design and PCR amplification. Gene blocks of V4-V5 sequences (Integrated DNA Technologies) were used as positive controls (Table S4). MEGA7 [34] was used to align target and non-target sequences and identify the most variable regions for primer design. We used Primer3 [35] to design primer sequences and calculate annealing conditions. Target specificity was checked against RDP Probe Match [36]. Target ASVs were quantified in all 60 samples of the JI time series using droplet digital PCR (ddPCR). Reactions were set up as follows: 11-l EvaGreen Supermix (Bio-Rad), 1.3-l 5-M forward and reverse primers, 6.4-l sterile water, and 2-l 100-diluted DNA template. PCR was run on a vapo-protect Mastercycler pro S under the following conditions: 95 C for 5 min; 40 cycles of 95 C for 30 s, 5860 C for 1 min; 4 C for 5 min; 90 C for 5 min; 4 C hold. The human *Bacteroides* (HB) marker, a human fecal marker in the genus *Bacteroides* [37], was quantified in the first 48 samples of the JI time series using qPCR, following methods described previously [38].

### Partitioning human-associated reads

We pulled Human Microbiome Project (HMP) sequence IDs and HTTP(S)/FTP links originating from studies 16S-PP1 and 16S-PP2 from the HMP resource page (https://hmpdacc.org/hmp/). HMP sequence IDs were uniquely de-replicated by their URL address. Batch-pulling of these sequence records resulted in a concatenated FASTA. A tool was created and applied to normalize metadata attributes across this dataset, followed by its subsequent reduction to unique sequences that occurred at least 10 times, with at least 1 subject and sample ID available for each uniquely filtered sequence. A parallelized, exact-identity sequence aligner was implemented and employed to align the sewage ASVs against the reduced HMP reference database, resulting in the parsing of human-associated sequences from the sewage dataset. We identified 491 human-associated ASVs within 35,332 total wastewater ASVs. For this study, the remaining ASVs were considered to be sequences from resident sewer microorganisms. Human-associated ASVs were binned by source body site (Table S5).

Due to sequencing errors and potential microorganism transfer among source environments, we established a threshold to identify and partition low-abundance, uncommon human-associated reads that were common in sewer samples (Figure S3). Among ASVs that were shared between WWTP influent and the HMP, if the minimum relative abundance across samples (5th-percentile) of a wastewater ASV exceeded the maximum relative abundance across samples (95th-percentile) of that ASV in the human microbiome, it was reclassified as a sewer-associated sequence. If the minimum abundance (5th-percentile) of a wastewater ASV was less than the maximum abundance (95th-percentile) of that ASV in the human microbiome, it was considered a human-associated sequence. After the filtering procedure, we moved 33 ASVs from a human-associated to a sewer-associated classification. In the final dataset, 458 ASVs in the wastewater samples were classified as human-associated.

### Statistics and graphics

We organized ASV and sample information with the R package phyloseq [39] (Table S6). The Shannon diversity index, a measure of alpha diversity, Bray-Curtis dissimilarity, a measure of beta diversity, and ordinations were calculated using the R package vegan [40]. We also used Mann-Whitney *U* tests, hierarchical clustering, autocorrelation function, linear regression, Shapiro-Wilk tests, and ANOVA to examine statistical relationships in the data, and these were performed using the R stats [41] package. We identified indicator ASVs with the R package indicspecies [42]. To reduce dataset complexity and examine the predominant bacteria, only ASVs with a maximum relative abundance of 1% or greater were considered in the indicator analyses. Principal coordinate analyses (PCoA) were conducted with the R package ape [43]. All figures were made in R with ggplot2 [44].

More specifically, we performed the following analyses to visualize and/or test statistically for differences in the community composition and abundance (qPCR/ddPCR) datasets: (1) a non-paired Mann-Whitney *U* test to compare Shannon diversity values between the US city and Milwaukee WWTP influent time-series datasets and the Milwaukee neighborhood and WWTP influent time-series datasets, 2) a non-paired Mann-Whitney *U* test to compare Bray-Curtis dissimilarity values between the US city and Milwaukee WWTP influent time-series datasets and the Milwaukee neighborhood and WWTP influent time-series datasets, (3) a Principle Coordinate Analysis (PCoA) of the Milwaukee time-series dataset to examine temporal patterns in community composition, (4) an indicator analysis (indicspecies [42]) to identify ASV relative abundance patterns that are indicative of groups of months, here set at exactly three consecutive month groupings in the Milwaukee time-series dataset, (5) a PERMANOVA test to identify if the month-based seasonal groupings of community composition are different statistically in the Milwaukee time-series dataset, (6) a correlation of environmental and sample metadata to the Bray-Curtis dissimilarity of bacterial community composition (envfit in R package vegan [40]) in the Milwaukee time-series dataset, (7) an examination of seasonality in abundance patterns of individual ASVs using hierarchical clustering of *z*-score normalized ASV relative abundances, where each ASV was relativized to its relative abundance values across the Milwaukee time-series samples, (8) the autocorrelation function (ACF) with 60 1-month time lags to test for data self-similarity with a defined time-lag; i.e., a test of significant seasonal patterns in the relative-abundance of particular ASVs, (9) Spearman rank correlations to test for relationships between ASV relative abundance data and the quantitative PCR data for select ASVs, and (10) a Mann-Whitney *U* test for seasonal differences in the US city community composition data (e.g., cold period northern city vs. warm period southern city). For more detailed information on the specific functions used, see Table S6 and our GitHub repository, https://github.com/NewtonLabUWM/Sewage_TimeSeries.

## Results

### Wastewater bacterial community diversity scales with time and space

**Fig. 1:**
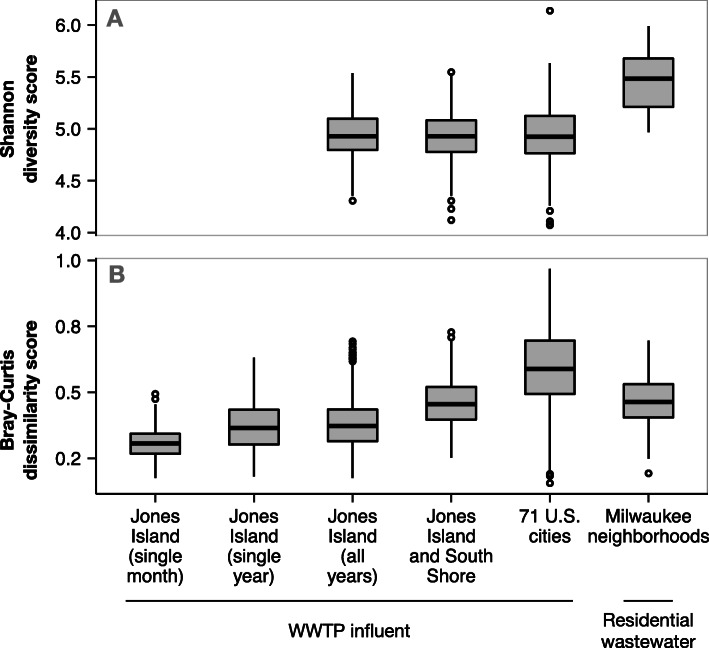
**a** Alpha diversity (measured with Shannon diversity), and **b** beta diversity (measured with Bray-Curtis dissimilarity) in raw wastewater bacterial communities. Diversity was measured in wastewater treatment plant influent in the 5-year time series of JI, in Milwaukee, WI; in JI and SS in Milwaukee, WI; from 77 WWTPs across the USA; and in wastewater collected from residential Milwaukee neighborhood sewers. Boxes depict the median and first and third quartiles. Whisker lines extend to interquartile ranges 1.5 and points are outlier values

The Shannon diversity index was similar between samples collected in the Milwaukee time series and US city WWTP influent datasets (Mann-Whitney *U*, *p* = 0.33; Fig. 1a), which indicates there is a relatively consistent number/evenness of bacterial taxa that co-inhabit these municipal sewer systems. In contrast, Shannon diversity was greater in the Milwaukee neighborhood wastewater samples than in the Milwaukee WWTP influent samples (Mann-Whitney *U*, *p* = 4.9 10^11^; Fig. 1a).

Contrasting the alpha diversity measure, the bacterial community composition was not similar across the WWTP influent datasets. Bray-Curtis dissimilarity increased as the sample set included more WWTPs or time points covering a greater proportion of a year in a single treatment plant (Fig. 1b). The range of Bray-Curtis dissimilarity values was similar between residential wastewater samples and the Milwaukee WWTP influent samples (Mann-Whitney *U*, *p* = 0.12) but was greater in the US city dataset (Mann-Whitney *U*, *p* < 2.2 10^16^). This result indicates that differences in environmental conditions among sewers have a larger influence on community composition than localized, within-system environmental differences.

### Resident sewer bacterial communities are distinct from the human microbiome

**Fig. 2:**
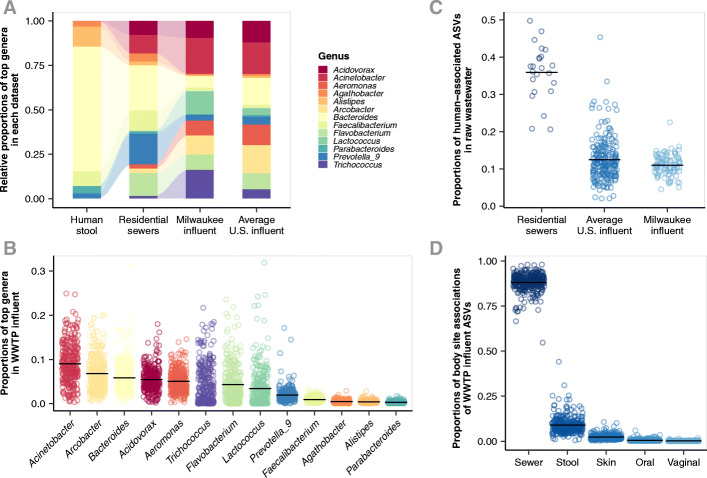
**a** Stacked bar plots showing the most abundant genera (top 5 from each dataset) among human stool samples from the Human Microbiome Project, wastewater from Milwaukee residential sewers, influent from a 5-year time series of two Milwaukee WWTPs, and WWTPs from across the US. Bar height indicates the proportion of that genus among the abundant genera visualized. Bar colors denotes the genus. **b** Proportion of abundant genera from Fig. 2a in all WWTP influent samples. c Proportion of all human-associated ASVs in the three wastewater datasets. d Proportion of ASVs from human microbiome body site sources among all wastewater samples. For bd, circles indicate sample value and lines indicate dataset mean

Bacteria associated with human stool became a lesser part of the overall community as wastewater traveled from neighborhood sewers to the WWTP (Fig. 2a). For example, *Bacteroides* was on average the most abundant genus in human stool (53% of community). It decreased to 11% in Milwaukee residential sewer communities and 3.4% in Milwaukee WWTP influent. *Acinetobacter* was the most abundant genus in WWTP influent communities in Milwaukee (11% of community) and across the US (8.8%). *Acinetobacter* was not as dominant in residential wastewater (5.3%) and was virtually absent (4.5 10^3^%) in human stool. Other abundant stool-associated genera, including *Alistipes*, *Faecalibacterium*, and *Parabacteroides*, also decreased in their contribution to the overall community as they moved from the human host, into the sanitary sewer system, and to the WWTP. Their dominance was replaced by genera not common to the human microbiome, such as *Arcobacter*, *Trichococcus*, and *Flavobacterium* (Fig. 2b).

The majority of wastewater bacteria were not associated with the human microbiome (Fig. 2c). In residential sewer communities, 35.9 7.5% of reads belonged to ASVs attributed to the human microbiome, but in the 5-year time series of two Milwaukee WWTPs, the proportion dropped to 11.0 2.8%. Similarly, across the USA, only 12.4 5.7% of reads were human-associated. Of the human microbiome sources, stool was the greatest contributor of ASVs to WWTP influent (9.0 4.7%; Fig. 2d). Overall, we find that the majority of reads in wastewater were assigned to ASVs that were not associated with the human microbiome (88.0 5.0%), and we considered them to be sewer-associated for subsequent analyses.

### Wastewater bacterial communities assemble into seasonal steady states

**Fig. 3:**
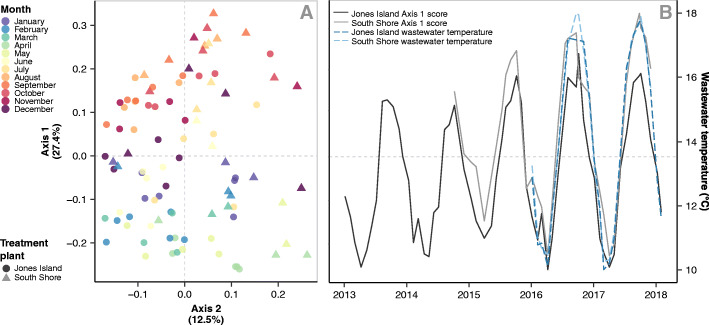
**a** Principal coordinate analysis (PCoA) of influent bacterial communities from two Milwaukee WWTPs sampled once a month for 5 years. Points indicate influent bacterial community samples, color denotes the month sampled, and shape indicates the source WWTP. Axis 1 is set as the *y*-axis for visualization purposes. **b** PCoA Axis 1 scores from both WWTPs over time (solid grey lines) plotted with wastewater temperatures (blue dashed lines)

Milwaukee WWTP influent bacterial communities repeatedly assembled into two community states each year (Fig. 3a), and the pattern was consistent for both of Milwaukees WWTP facilities (Fig. 3b). In a PCoA, all samples from January through May had Axis 1 scores less than 0, while samples from August through November had Axis 1 scores greater than 0. Samples from June, July, and December had both positive and negative Axis 1 scores. Typically, samples from April to May and September to October harbored the most distinct community compositions (Fig. 3a).

We conducted an indicator analysis to identify ASVs that had relative abundance patterns that were indicative of chronologic 3-month groups. Only ASVs with a maximum relative abundance 1% were considered. With this analysis we identified 14 indicator ASVs (Table S7). The indicator results also supported the monthly groupings of the PCoA, as we only found indicators of month groups including February through June and August through December (Table S7). No indicator ASVs were found for 3-month groups containing July or January, suggesting these are periods of transition between community types. For this reason, we described wastewater from February through June as the spring steady state and wastewater from August to December as the fall steady state, with the primary differentiating months being FebruaryMay and AugustNovember. We also assessed the statistical strength of these month-based community groupings with a PERMANOVA test on the Bray-Curtis distance matrix for the following groups: (1) spring = February to June, (2) fall = August to December, and (3) mix = January and July. The PERMANOVA test also supported the idea of these months having distinct bacterial communities (*R*^2^ = 0.212, *p* = 0.0099).

Wastewater temperature was very tightly coupled to the change in community composition at both Milwaukee WWTPs (environmental fit, JI *R*^2^ = 0.96, SS *R*^2^ = 0.97; Fig. 3b) and appears to be the primary driver of the observed seasonal change in community composition. For the other measured environmental parameters, we saw differences between the WWTPs in their relationship to bacterial community composition. At SS, which receives only wastewater from a separated sewer system, all variables tested (flow rate, ammonia, total suspended solids, air temperature, phosphorus, biological oxygen demand, precipitation, and year) were significant predicators (environmental fit *R*^2^ range = 0.350.85) of influent bacterial communities (Table S8), but were less strongly related to community change than temperature. At JI, which receives combined sewer wastewater, the environmental parameters measured were much less indicative of the community composition (environmental fit, *R*^2^ range *R*^2^ range = 0.00350.28; Table S8).

### Sewer bacteria drive temporal trends in WWTP influent

**Fig. 4:**
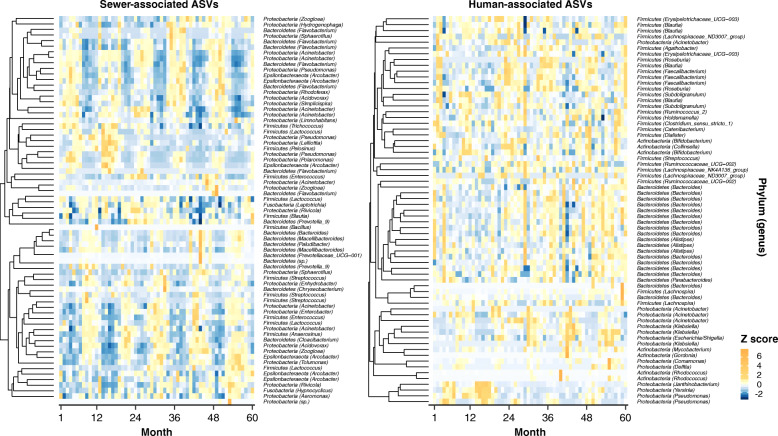
Dendrograms and heatmaps of abundant (maximum relative abundance > 1%) sewer-associated (left) and human-associated (right) ASVs in a 5-year time series of JI influent. Heatmap colors denote within-ASV *Z*-scored normalized relative abundances

Seasonal bacterial community variation was driven more by abundance changes of common sewer-associated ASVs than by human-associated ASVs. Dendrogram clustering of normalized sewer-associated ASV abundances (Milwaukee time-series) illustrated that many common wastewater bacteria (e.g., *Acinetobacter*, *Arcobacter*, *Cloacibacterium*, *Flavobacterium*, *Lactococcus*) exhibited repeating temporal patterns of high/low or low/high abundance in the spring and fall states (Fig. 4). In contrast to the seasonal abundance pattern clustering of sewer-associated ASVs in the influent samples, common human ASVs exhibited less dramatic temporal fluctuations, and these changes were not predictable temporally or with the wastewater environmental data. Instead, human ASV relative abundance patterns often clustered by taxonomic affiliation (Fig. 4).

**Fig. 5:**
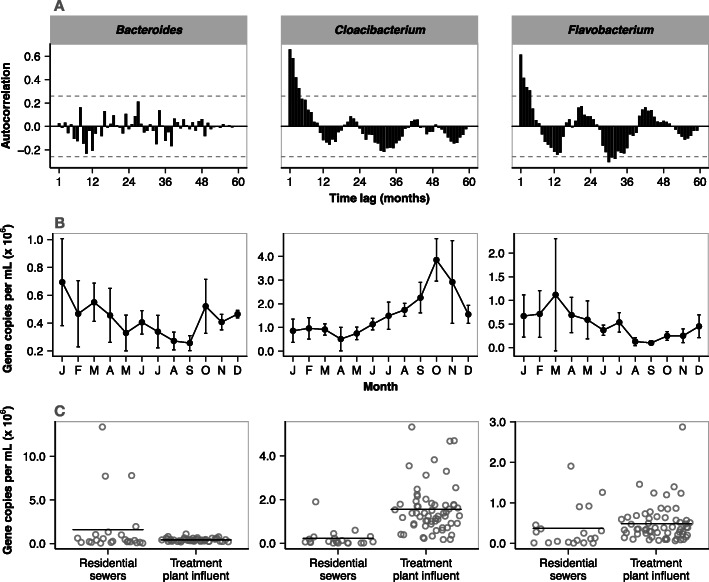
**a** Time-dependent autocorrelations of relative abundance change for select ASVs performed with 60 1-month time lags from the JI wastewater influent dataset. Bar height indicates autocorrelation score at each time lag, and grey dashed lines indicate autocorrelation significance level ( 0.26 at *p* = 0.05). **b** Line graph of quantitative PCR measurements targeting these ASVs in JI influent. Vertical lines extend to standard deviations. (C) Quantitative PCR measurements in Milwaukee neighborhood sewers and JI influent. Horizontal lines indicate mean gene concentration. Taxonomic affiliation of the ASVs includes (left) a human-specific *Bacteroides*; (middle) a fall-associated, sewer-specific *Cloacibacterium*; and (right) a spring-associated, sewer-specific *Flavobacterium*

We identified two sewer-associated ASVs that exhibited significant seasonal abundance variation and one ASV matching a human fecal indicator that did not. The two sewer organisms were (1) ASV8, an indicator of SeptemberOctoberNovember (fallwarm period) classified to the genus *Cloacibacterium*; and (2) ASV42, an indicator of FebruaryMarchApril (springcold period) classified as *Flavobacterium*. The human fecal indicator was classified as a *Bacteroides* (ASV44). This ASV has 100% sequence identity to the Human Bacteroides marker, a well-established marker for tracking human fecal pollution in the environment [37]. We ran autocorrelation function (ACF) with 60 1-month time lags to verify the observed seasonal relative abundance patterns of the *Cloacibacterium* and *Flavobacterium* ASVs. The autocorrelation function confirmed the repeated seasonal cycle for these two ASVs across the 5-year time series (Fig. 5a). The human specific *Bacteroides* did not show significant autocorrelations (*p* value = 0.05) at any time lag (Fig. 5a).

ASV-specific gene quantifications demonstrated relative abundance patterns observed in the sequence-based datasets translated to actual abundance change. The *Cloacibacterium* ASV had the highest relative and absolute abundance ranking in WWTP influent (1.1 0.69%, 1.5 10^6^ 1.1 10^6^ copies/ml), followed by the *Flavobacterium* (0.41 0.25%) and *Bacteroides* (0.21 0.07%) ASVs (Fig. 5c). Absolute abundance quantification matched relative abundance patterns for *Cloacibacterium* (Spearman rank correlation, rho = 0.85; Fig. 5b), *Flavobacterium* (rho = 0.83) and *Bacteroides* (rho = 0.49). These measurements also support the observation that water temperature drives fluctuations in the resident sewer bacterial community, in that *Cloacibacterium* and *Flavobacterium* concentrations were correlated to wastewater temperature (Spearman rank correlation, rho = 0.90 and 0.89, respectively; *p* = 2.810^9^ and 6.4 10^9^, respectively), while *Bacteroides* concentrations were not (rho = 0.22, *p* = 0.30).

### Milwaukee wastewater seasonality is supported spatially across the USA

**Fig. 6:**
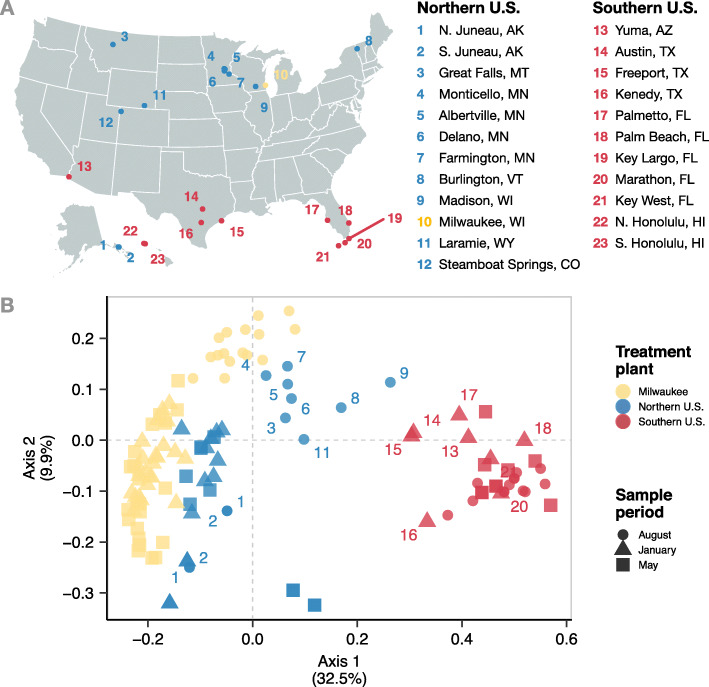
Top: map of select wastewater treatment plants sampled previously [27]. Bottom: principal coordinate analysis of influent bacterial communities. Yellow points indicate samples from the 5-year Milwaukee WWTP time series, blue points the 11 coldest US cities in the dataset, and red points the 11 warmest US cities in the dataset. Point shapes indicate the sampling period during which wastewater was collected. Points with labels are samples from either southern US cities in January or northern US cities in August

Northern and southern US cities had distinct bacterial WWTP influent communities (Fig. 6). Seasonal change altered the magnitude of this regional community composition difference. For example, communities in northern cities (a cold region) during August (a high temperature period) were more similar to communities from southern US cities (a warm region) than they were to other northern communities when it was cold (Mann-Whitney *U*, *p* < 2.2 10^16^; Figure S4). This similarity was greatest when southern cities experienced their coldest temperatures. Also, southern US cities, which experience less dramatic seasonal temperature change, had WWTP influent communities that were less variable than the northern cities (Figure S5).

## Discussion

Wastewater conveyance represents a unique ecosystem in urban environments. Sewers maintain a resident community of microorganisms, while transient organisms continuously wash in from urban waste, runoff, and the human microbiome. In a study of a single WWTP, human gut microorganisms represented a relatively small fraction of the influent community (~ 7%) [26]. Our work across dozens of facilities supports this observation; we observe roughly 1015% of the community is human-derived. The wastewater community also changes in relation to its location in the system. Human-derived microorganisms represent a larger fraction (~ 36%) of the community up-the-pipe (i.e., neighborhood sewers), but as wastewater flows through the system, resident bacteria become dominant, reaching > 85% of the assemblage. We believe this shift results from a significant increase in resident sewer bacteria, rather than a decay in human-associated microorganisms during transit. In our relatively limited testing, we found resident sewer organisms increased 2.7- to 19-fold from neighborhood sewers to the treatment plant, while human-associated bacteria stayed relatively constant (1.3-fold change).

We note that we did not attempt to partition what we term the resident community into organisms washing in from urban waste versus those that are truly sewer residents. Others have suggested soil bacteria may make up a significant fraction (> 20%) of sewer microbes [26]. We agree that this is likely, but it is not clear if these organisms are sewer residents having originating from soil or represent transient flux into the system. Truly transient sewer organisms should have highly variable distributions in time, and nearly all of the abundant organisms in our defined resident fraction were present consistently. More work is needed to further identify the true permanent sewer residents and the possible origins for these residents. Understanding these details would contribute to both the development of markers for sewage pollution tracking [45, 46] and further the understanding of which organisms are universally present and thus likely metabolically active inside these pipes.

There are numerous places in conveyance systems that can accumulate high concentrations of actively growing sewer microbes. Biofilms attached to interior pipe surfaces represent one potentially large reservoir of resident organisms, and several studies have examined these communities (reviewed by Li et al. 2019 [47]). Community compositions of sewer pipe biofilm and WWTP influent suggest there is considerable interactions between the two environments, but additional sewer habitats, such as sediments, may be contributing even larger microbial loads to the wastewater [6]. An already significant effort has been put forth to understand the products of sewer biofilm activity [47], as concrete corrosion from these activities costs more than $1 billion globally each year [48]. More work is needed in a single system to eliminate cross-system variability so that unique habitats can be identified and described.

Predominant sewer microorganisms were consistent across all the systems we examined, and also seem to be common in systems globally [6, 26]. Although the same genera are present, there are stark differences in the actual bacterial composition among sewer systems, and clear diversity patterns similar to those found in other aquatic ecosystems systems like lakes or the ocean. We found that alpha diversity in WWTP influent samples remains relatively constant, but up-the-pipe, the diversity was often greater. Because human microbiome contributions were greater up-the-pipe, individual variations in these samples and household waste streams presumably increases this diversity, but this remains to be tested. Meanwhile, we believe the large, integrated water network of conveyance systems homogenizes community inputs, obscuring rarer members prior to sampling of WWTP influent. We also found that beta diversity of influent increases as more sites or more times of the year are included, but not as more years are included. This pattern is very similar to the seasonal river, lake, or oceanic basin microbial community patterns where communities predictably cycle each year, but each system has its own unique community structure and timing of community change [22, 49,51].

Our time series revealed sewer resident communities exhibit significant and repeatable temporal community change, which manifested as a seasonal cycle. This was surprising to us, as surface-water seasonal cycles such as those in temperate lakes are driven by changes in a combination of temperature and light availability, which influence primary production and ultimately start a cascade of change through the food web [52]. Sewers are below-ground and thus are buffered to large temperature changes (e.g., in Milwaukee ~ 8 C difference across a year), no light is available, and there is constant exogenous nutrient inputs, so it appears much of the seasonal regime is tied to wastewater temperature change. Indeed, in Milwaukee, the seasonality of the influent wastewater bacterial community composition at both treatment plants (JI and SS) was clearly driven by wastewater temperature. Some of the other measured physical-chemical parameters also correlated to the community change (48-h precipitation, flow, ammonia, BOD, total phosphorus, TSS; Table S8), but the majority of these relationships were significant at only one of the two treatment plants (the SS plant, a separated sewer system), and the correlations were weaker than that found for water temperature. To us, it is clear that temperature is a primary driver of bacterial community change in at least some wastewater conveyance systems.

In Milwaukee, sewer wastewater temperature change lags behind the change in air temperature, resulting in a roughly 3-month delay between the lowest/highest average air temperatures and the lowest/highest temperatures in the wastewater. This results in the bacterial community composition being most distinct at the wastewater temperature extremes, which occur in April (cold, ~ 10 C) and October (warm, ~ 18 C). Although we do not have long-term time series data from other cities, it appears water temperature plays a primary role in structuring and geographically partitioning sewer bacterial communities across vast geographical distances. Communities from northern (cold) and southern (warm) US cities were strikingly distinct, but they became more similar in comparisons of warm periods in the north to cold periods in the south. The regional warm periods in the north and cold periods in the south occur asynchronously, so there is no apparent period of community convergence across these distinct temperature regions. Also, we do not have seasonal wastewater temperatures for any southern cities, so it is unknown if the pace and timing of community change in these systems matches the two-season (warm-fall to cold-spring) setup observed in the Milwaukee dataset. It also appears that southern US cities, which have smaller air temperature ranges than most northern cities, have correspondingly less variable bacterial communities. We presume these two conditions are related, but the question of how the magnitude of wastewater temperature change impacts community composition remains to be tested.

## Conclusions

Temperature dependence is clearly driving large-scale changes to the bacterial community composition in municipal wastewater conveyance systems. The temperature change results in a bacterial community that exhibits striking seasonality, but this seasonal cycle occurs in a below-ground and built/engineered system. Seasonality is more typically described for surface communities, which experience both light and temperature changes over a year. This community pattern indicates the microbial communities in built infrastructure have emergent properties comparable to the rest of the aquatic microbial biosphere; and therefore, further examination of how these microbial communities adapt to built water infrastructure is warranted. Going forward, it also needs to be determined whether temperature-driven cycles in wastewater impact engineering processes at treatment plants or alter sewer pipe corrosion rates. Wastewater treatment plant performance can vary seasonally, but it is still unclear how much of this is driven by changes in the entering community. Additionally, seasonal change in wastewater communities may represent a change in the levels of human or environmental health risk during untreated sewage release. Although the human fecal bacteria remain fairly constant temporally, the seasonal abundance shifts for common sewer organisms could be used to develop more sensitive seasonal or regional specific indicators for sewage pollution tracking. Overall, we advocate for applying microbial ecological theory developed from natural ecosystems to sewer systems. Much like in the relatively new discipline of urban ecology, there are likely theories that apply across natural and built system boundaries, but also unique paradigms that exist only in the built systems. Sewers allow for some operational control and thus could prove useful in testing theories across boundaries, but also for understanding how urban environments alter microbial community assembly, activity, and adaptation.

## Supplementary Information

**Additional file 1: Figure S1.** Map of wastewater treatment plants (WWTPs) sampled across the US. **Table S1.** Information for WWTPs sampled across the US. **Figure S2.** Map of residential neighborhood manholes sampled in Milwaukee, WI. **Table S2.** Information for manholes sampled in Milwaukee, WI. **Table S3.** Primer sequences targeting sewer-associated *Cloacibacterium* and *Flavobacterium.* **Table S4.** Gene block sequences of 16S rRNA V4-V5 gene amplicons. **Table S5.** Schema used to bin HMP body site descriptions into simpler terms. **Figure S3.** Diagram showing logic of microbiome classification threshold. **Table S6.** R packages & datasets used for analysis. **Table S7.** Indicator ASVs in three-month windows of the Jones Island time series. **Table S8.** Scores from fitting microbial communities to environmental data. **Figure S4.** Bray-Curtis dissimilarity measurements between the Southern US, Northern US, and time series. **Figure S5.** Heights from cluster analysis of the Southern US, Northern US, and the time series.

**Additional file 2.**

## Data Availability

The datasets generated and analyzed during the current study are available in our GitHub repository, https://github.com/NewtonLabUWM/Sewage_TimeSeries.
