# Supplementary material for: Urban wastewater bacterial communities assemble into seasonal steady states
Source: Microbiome. 2021 May 20;9:116. doi: 10.1186/s40168-021-01038-5 (PMC8139061; doi:10.1186/s40168-021-01038-5)
Supplement: Supplementary file 2 — Additional file 1: Figure S1. Map of wastewater treatment plants (WWTPs) sampled across the US. Table S1. Information for WWTPs sampled across the US. Figure S2. Map of residential neighborhood manholes sampled in Milwaukee, WI. Table S2. Information for manholes sampled in Milwaukee, WI. Table S3. Primer sequences targeting sewer-associated Cloacibacterium and Flavobacterium. Table S4. Gene block sequences of 16S rRNA V4-V5 gene amplicons. Table S5. Schema used to bin HMP body site descriptions into simpler terms. Figure S3. Diagram showing logic of microbiome classification threshold. Table S6. R packages & datasets used for analysis. Table S7. Indicator ASVs in three-month windows of the Jones Island time series. Table S8. Scores from fitting microbial communities to environmental data. Figure S4. Bray-Curtis dissimilarity measurements between the Southern US, Northern US, and time series. Figure S5. Heights from cluster analysis of the Southern US, Northern US, and the time series. [file 40168_2021_1038_MOESM2_ESM.docx]

**Supporting information:**

Urban wastewater bacterial communities assemble into seasonal steady states

Emily L. LaMartina, Aurash A. Mohaimani^†^, Ryan J. Newton*

School of Freshwater Sciences, University of Wisconsin-Milwaukee, Milwaukee, WI, 53204 United States

†Present Address: Analytical Technologies, Biogen, 5000 Davis Dr. Morrisville, NC, US

* Corresponding Author: newtonr@uwm.edu

**TABLE OF CONTENTS**

| **Figure S1** | Map of wastewater treatment plants (WWTPs) sampled across the US | S1 |
| --- | --- | --- |
| **Table S1** | Information for WWTPs sampled across the US | S2 |
| **Figure S2** | Map of residential neighborhood manholes sampled in Milwaukee, WI | S3 |
| **Table S2** | Information for manholes sampled in Milwaukee, WI | S4 |
| **Table S3** | Primer sequences targeting sewer-associated *Cloacibacterium* and *Flavobacterium* | S5 |
| **Table S4** | Gene block sequences of 16S rRNA V4-V5 gene amplicons | S6 |
| **Table S5** | Schema used to bin HMP body site descriptions into simpler terms | S7 |
| **Figure S3** | Diagram showing logic of microbiome classification threshold | S8 |
| **Table S6** | R packages & datasets used for analysis | S9 |
| **Table S7** | Indicator ASVs in three-month windows of the Jones Island time series | S14 |
| **Table S8** | Scores from fitting microbial communities to environmental data | S15 |
| **Figure S4** | Bray-Curtis dissimilarity measurements between the Southern US, Northern US, and time series | S16 |
| **Figure S5** | Heights from cluster analysis of the Southern US, Northern US, and the time series | S17 |

**
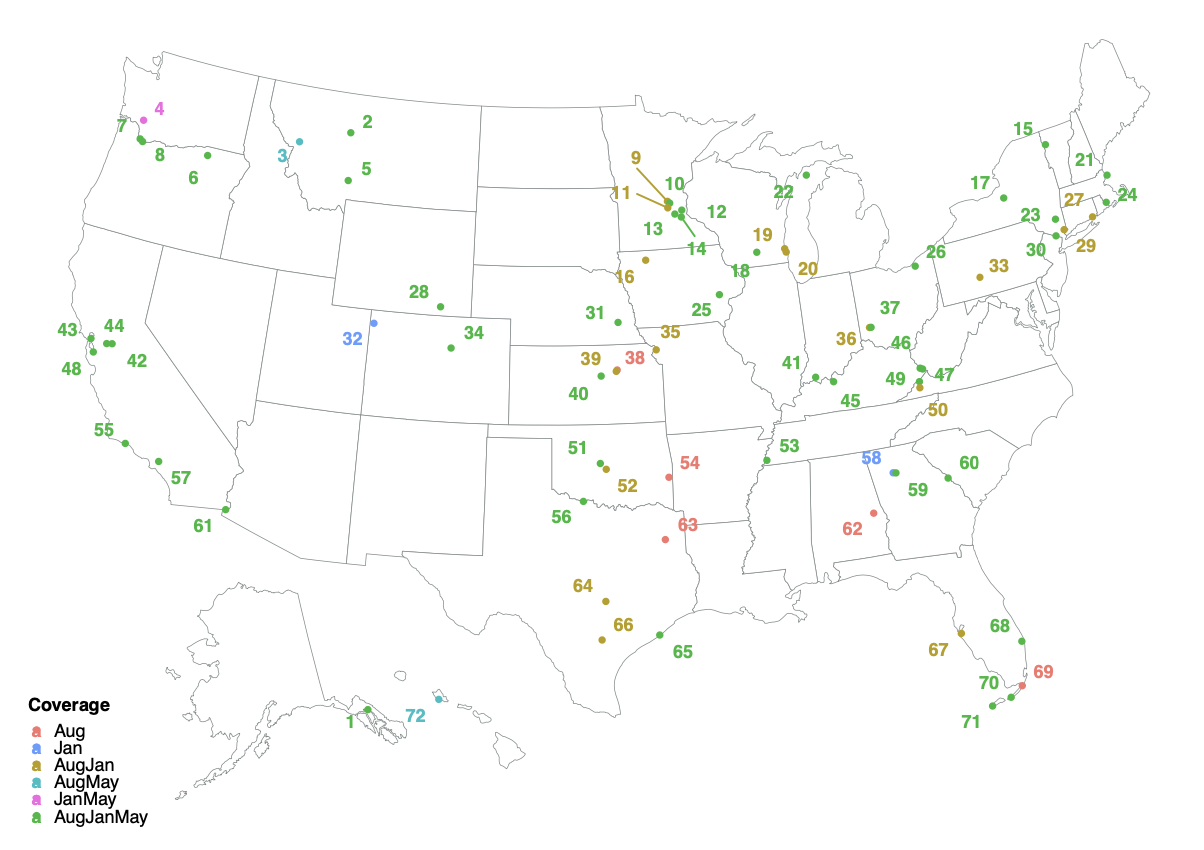
Figure S1.** Map of wastewater treatment plants (WWTPs) sampled across the US for Newton et al., 2015. WWTP influent was collected during three sampling periods designated as August, January, and May. Because influent was not collected from all three periods at each WWTP, “coverage” indicates which sampling periods were collected at each location.

Newton, R. J., McLellan, S. L., Dila, D. K., Vineis, J. H., Morrison, H. G., Murat Eren, A., Sogin, M. L. Sewage Reflects the Microbiomes of Human Populations. *mBio* **2015**, *6* (2), 1–9. https://doi.org/10.1128/mBio.02574-14.

**Table S1.** Information for WWTPs sampled across the US for Newton et al., 2015. Label and coverage corresponds to Figure S1.

| **Label** | **Longitude** | **Latitude** | **State** | **City** | **Coverage** | **Label** | **Longitude** | **Latitude** | **State** | **City** | **Coverage** |
| --- | --- | --- | --- | --- | --- | --- | --- | --- | --- | --- | --- |
| **1** | -134.385 | 58.286 | Alaska | Juneau | AugJanMay | **37** | -84.24 | 39.55 | Ohio | Springboro | AugJanMay |
| **2** | -111.299 | 47.521 | Montana | Great Falls | AugJanMay | **38** | -96.819 | 39.044 | Kansas | Junction City East | Aug |
| **3** | -114.042 | 46.88 | Montana | Missoula | AugMay | **39** | -96.866 | 38.992 | Kansas | Junction City | AugJan |
| **4** | -122.73 | 46.324 | Washington | Kelso | JanMay | **40** | -97.607 | 38.833 | Kansas | Salina | AugJanMay |
| **5** | -111.069 | 45.723 | Montana | Bozeman | AugJanMay | **41** | -87.289 | 38.053 | Indiana | Boonville | AugJanMay |
| **6** | -118.837 | 45.668 | Oregon | Pendleton | AugJanMay | **42** | -121.328 | 37.936 | California | Stockton | AugJanMay |
| **7** | -122.626 | 45.617 | Washington | Vancouver | AugJanMay | **43** | -122.378 | 37.919 | California | Richmond | AugJanMay |
| **8** | -122.458 | 45.547 | Oregon | Portland | AugJanMay | **44** | -121.584 | 37.889 | California | Discovery Bay | AugJanMay |
| **9** | -93.775 | 45.298 | Minnesota | Monticello | AugJan | **45** | -86.469 | 37.78 | Kentucky | Hardinsburg | AugJanMay |
| **10** | -93.661 | 45.225 | Minnesota | Albertville | AugJanMay | **46** | -82.275 | 37.67 | West Virginia | Williamson | AugJanMay |
| **11** | -93.784 | 45.052 | Minnesota | Delano | AugJan | **47** | -82.166 | 37.621 | West Virginia | Matewan | AugJanMay |
| **12** | -93.046 | 44.924 | Minnesota | St. Paul | AugJanMay | **48** | -122.111 | 37.453 | California | Palo Alto | AugJanMay |
| **13** | -93.431 | 44.798 | Minnesota | Shakopee | AugJanMay | **49** | -82.431 | 37.18 | Virginia | Clintwood | AugJanMay |
| **14** | -93.104 | 44.666 | Minnesota | Farmington | AugJanMay | **50** | -82.467 | 36.951 | Virginia | Coeburn | AugJan |
| **15** | -73.22 | 44.472 | Vermont | Burlington | AugJanMay | **51** | -97.752 | 35.525 | Oklahoma | Yukon | AugJanMay |
| **16** | -95.132 | 43.136 | Iowa | Spencer | AugJan | **52** | -97.485 | 35.298 | Oklahoma | Moore | AugJan |
| **17** | -76.178 | 43.064 | New York | Syracuse | AugJanMay | **53** | -90.055 | 35.188 | Tennessee | Memphis | AugJanMay |
| **18** | -89.357 | 43.036 | Wisconsin | Madison | AugJanMay | **54** | -94.616 | 34.885 | Oklahoma | Heavener | Aug |
| **19** | -87.9 | 43.022 | Wisconsin | Milwaukee | AugJan | **55** | -119.686 | 34.417 | California | Santa Barbara | AugJanMay |
| **20** | -87.846 | 42.888 | Wisconsin | Oak Creek | AugJan | **56** | -98.562 | 34.102 | Texas | Burkburnett | AugJanMay |
| **21** | -70.68 | 42.611 | Massachusetts | Gloucester | AugJanMay | **57** | -118.023 | 34.034 | California | Whittier | AugJanMay |
| **22** | 71.006 | 42.047 | Massachusetts | Brockton | AugJanMay | **58** | -84.392 | 34.002 | Georgia | Roswell | Jan |
| **23** | -73.936 | 41.717 | New York | Poughkeepsie | AugJanMay | **59** | -84.267 | 33.977 | Georgia | Johns Creek | AugJanMay |
| **24** | -71.193 | 41.676 | Massachusetts | Fall River | AugJanMay | **60** | -81.976 | 33.395 | Georgia | Augusta | AugJanMay |
| **25** | -91.506 | 41.611 | Iowa | Iowa City | AugJanMay | **61** | -114.664 | 32.73 | Arizona | Yuma | AugJanMay |
| **26** | -81.475 | 41.456 | Ohio | Woodmere | AugJanMay | **62** | -85.544 | 32.627 | Alabama | Auburn | Aug |
| **27** | -72.097 | 41.343 | Connecticut | New London | AugJan | **63** | -94.932 | 32.533 | Texas | Gladewater | Aug |
| **28** | -105.605 | 41.338 | Wyoming | Laramie | AugJanMay | **64** | -97.651 | 30.279 | Texas | Austin | AugJan |
| **29** | -73.677 | 41.243 | New York | Bedford | AugJan | **65** | -95.378 | 28.917 | Texas | Freeport | AugJanMay |
| **30** | -74.164 | 41.12 | New York | Hillburn | AugJanMay | **66** | -97.849 | 28.814 | Texas | Kenedy | AugJan |
| **31** | -96.688 | 40.839 | Nebraska | Lincoln | AugJanMay | **67** | -82.596 | 27.526 | Florida | Palmetto | AugJan |
| **32** | -108.858 | 40.496 | Colorado | Steamboat Springs | Jan | **68** | -80.132 | 26.741 | Florida | West Palm Beach | AugJanMay |
| **33** | -78.426 | 40.456 | Pennsylvania | Duncansville | AugJan | **69** | -80.448 | 25.086 | Florida | Key Largo | Aug |
| **34** | -104.956 | 39.808 | Colorado | Denver | AugJanMay | **70** | -80.983 | 24.746 | Florida | Marathon | AugJanMay |
| **35** | -94.866 | 39.731 | Missouri | St. Joseph | AugJan | **71** | -81.797 | 24.569 | Florida | Key West | AugJanMay |
| **36** | -84.319 | 39.553 | Ohio | Franklin | AugJan | **72** | -158.037 | 21.331 | Hawaii | Honolulu | AugMay |

**
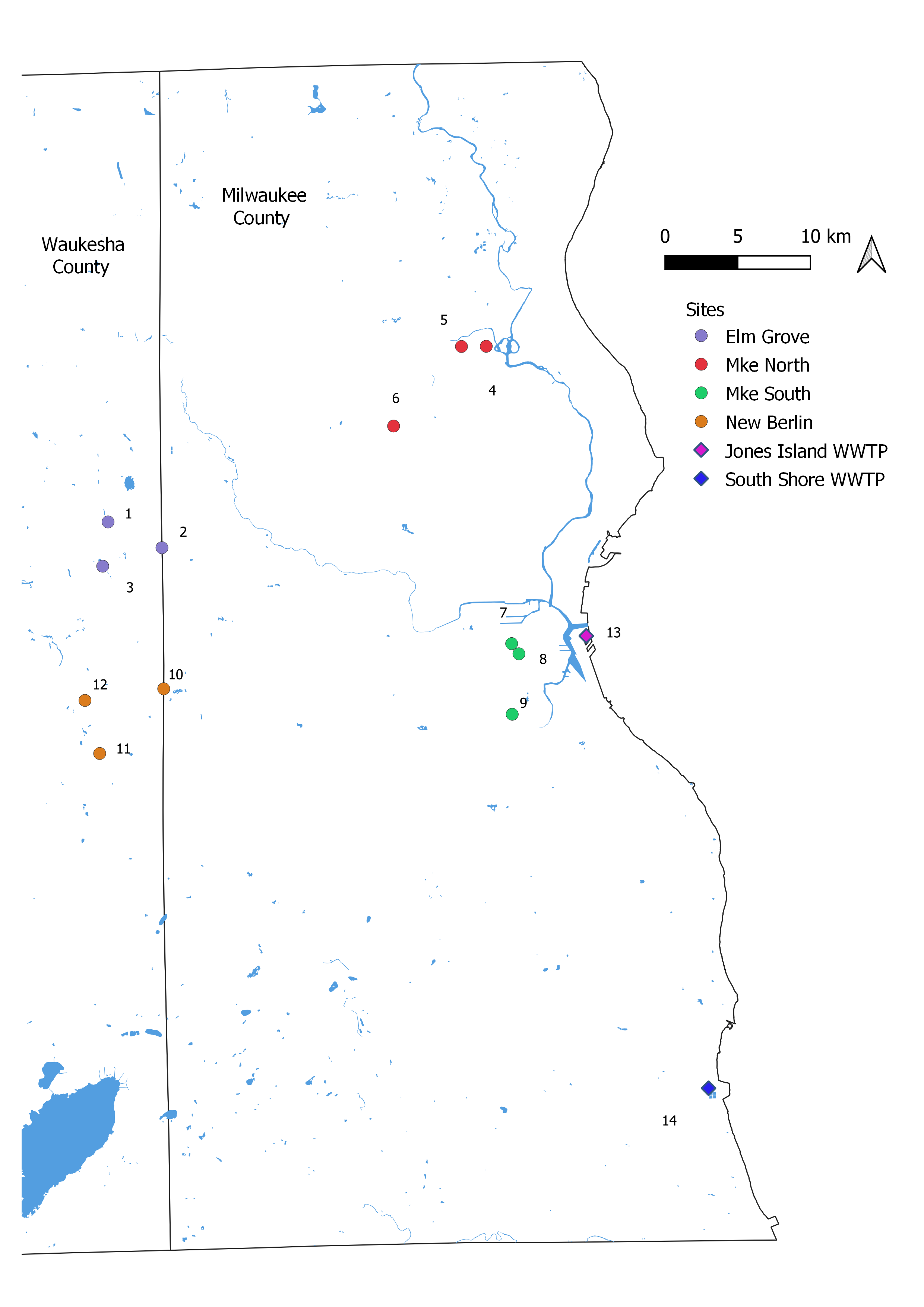
**

**Figure S2.** Map of sewer access in neighborhoods where raw wastewater was collected (ArcGIS map courtesy of Emily R. Koster).

**Table S2.** Location information of sewer access in neighborhoods where raw wastewater was collected.

| **Sample name** | **Collection date** | **Location ID** | **Neighborhood** | **Latitude** | **Longitude** | **Manhole depth** | **Pipe Diameter** | **Approx. # of households in drainage area** |
| --- | --- | --- | --- | --- | --- | --- | --- | --- |
| EG0001_15 | 12/15/15 | EG0001 | Elm Grove | 43.059194 | -88.088417 | 12.5 | 12 | 202 |
| EG0001_16 | 12/16/15 | EG0001 | Elm Grove | 43.059194 | -88.088417 | 12.5 | 12 | 202 |
| EG0002_15 | 12/15/15 | EG0002 | Elm Grove | 43.051197 | -88.066911 | 12 | 10 |  |
| EG0002_16 | 12/16/15 | EG0002 | Elm Grove | 43.051197 | -88.066911 | 12 | 10 |  |
| EG0003_15 | 12/15/15 | EG0003 | Elm Grove | 43.046139 | -88.090972 | 12.75 | 8 |  |
| EG0003_16 | 12/16/15 | EG0003 | Elm Grove | 43.046139 | -88.090972 | 12.75 | 8 |  |
| MINORTH1_15 | 12/15/15 | MINORTH1 | Mke North | 43.108488 | -87.934094 | 16.5 | 15 |  |
| MINORTH1_16 | 12/16/15 | MINORTH1 | Mke North | 43.108488 | -87.934094 | 16.5 | 15 |  |
| MINORTH2_15 | 12/15/15 | MINORTH2 | Mke North | 43.108618 | -87.944102 | 11 | 10 |  |
| MINORTH2_16 | 12/16/15 | MINORTH2 | Mke North | 43.108618 | -87.944102 | 11 | 10 |  |
| MINORTH3_15 | 12/15/15 | MINORTH3 | Mke North | 43.085571 | -87.972281 | 11.75 | 12 |  |
| MINORTH3_16 | 12/16/15 | MINORTH3 | Mke North | 43.085571 | -87.972281 | 11.75 | 12 |  |
| MISOUTH1_15 | 12/15/15 | MISOUTH1 | Mke South | 43.020373 | -87.926796 | 11.25 | 24 |  |
| MISOUTH1_17 | 12/17/15 | MISOUTH1 | Mke South | 43.020373 | -87.926796 | 11.25 | 24 |  |
| MISOUTH2_15 | 12/15/15 | MISOUTH2 | Mke South | 43.017306 | -87.923972 | 11 | 30 |  |
| MISOUTH2_17 | 12/17/15 | MISOUTH2 | Mke South | 43.017306 | -87.923972 | 11 | 30 |  |
| MISOUTH3_15 | 12/15/15 | MISOUTH3 | Mke South | 42.999472 | -87.927278 | 14 | 21 |  |
| MISOUTH3_17 | 12/17/15 | MISOUTH3 | Mke South | 42.999472 | -87.927278 | 14 | 21 |  |
| NB0001_15 | 12/15/15 | NB0001 | New Berlin | 43.009472 | -88.0675 | 12 | 18 | 625 |
| NB0001_16 | 12/16/15 | NB0001 | New Berlin | 43.009472 | -88.0675 | 12 | 18 | 625 |
| NB0002_15 | 12/15/15 | NB0002 | New Berlin | 42.990768 | -88.093917 | 11.3 | 8 |  |
| NB0002_16 | 12/16/15 | NB0002 | New Berlin | 42.990768 | -88.093917 | 11.3 | 8 |  |
| NB0003_15 | 12/15/15 | NB0003 | New Berlin | 43.006556 | -88.099361 | 11.5 | 8 |  |
| NB0003_16 | 12/16/15 | NB0003 | New Berlin | 43.006556 | -88.099361 | 11.5 | 8 |  |

**Table S3.** Primer sequences and PCR assay conditions targeting sewer-associated *Cloacibacterium* (ASV8) and *Flavobacterium* (ASV42).

| **Target** | **Primer name^1^** | **Direction** | **Primer sequence** | **Anneal temp (**°C) |
| --- | --- | --- | --- | --- |
| *Flavobacterium* ASV11^2^ | flavo11_sew_629F | Forward | GAACGGCCATTGATACTGCT | 58-60 |
|  | flavo11_sew_859R | Reverse | TAGCCACTGAAGTTGCCCCC |  |
| *Flavobacterium* ASV42 | flavo42_sew_630F | Forward | ACGGCCATTGATACTGTCTGA | 58-60 |
|  | flavo42_sew_859R | Reverse | TAGCCACTGAGATTGCTCCC |  |
| *Cloacibacterium* ASV8 | cloaci08_sew_682F | Forward | AGTGTAGCGGTGAAATGCAT | 58-60 |
|  | cloaci08_sew_860R | Reverse | TTGGTCTCTGAACCCTAAAGC |  |
| *Cloacibacterium* ASV32^2^ | cloaci32_sew_859R^3^ | Reverse | TGGTCTCTGAAGCTTGCGCT | 58-60 |

^1^Primer names include genus name, ASV number, source, and *E. coli* 16S rRNA gene position.

^2^Sequences representing *Flavobacterium* ASV11 and *Cloacibacterium* ASV32 served as negative controls for the target assays.

^3^ASV8 and ASV32 share the same forward primer.

**Table S4.** Gene blocks of 16S rRNA V4-V5 gene amplicon sequences.

| **Gene block name** | **16S rRNA gene V4-V5 sequence** |
| --- | --- |
| flavo11_sew_16S | ACGGAGGATCCAAGCGTTATCCGGAATCATTGGGTTTAAAGGGTCCGTAGGCGGTTTAGTAAGTCAGTGGTGAAAGCCCATCGCTCAACGGTGGAACGGCCATTGATACTGCTAGACTTGAATTATTAGGAAGTAACTAGAATATGTAGTGTAGCGGTGAAATGCTTAGAGATTACATGGAATACCAATTGCGAAGGCAGGTTACTACTAATGGATTGACGCTGATGGACGAAAGCGTGGGTAGCGAACAGGATTAGATACCCTGGTAGTCCACGCCGTAAACGATGGATACTAGCTGTTGGGGGCAACTTCAGTGGCTAAGCGAAAGTGATAAGTATCCCACCTGGGGAGTACGTTCGCAAGAATGAA |
| flavo42_sew_16S | ACGGAGGATCCAAGCGTTATCCGGAATCATTGGGTTTAAAGGGTCCGTAGGCGGTCAGATAAGTCAGTGGTGAAAGCCCATCGCTCAACGGTGGAACGGCCATTGATACTGTCTGACTTGAATTATTAGGAAGTAACTAGAATATGTAGTGTAGCGGTGAAATGCTTAGAGATTACATGGAATACCAATTGCGAAGGCAGGTTACTACTAATGGATTGACGCTGATGGACGAAAGCGTGGGTAGCGAACAGGATTAGATACCCTGGTAGTCCACGCCGTAAACGATGGATACTAGCTGTTGGGAGCAATCTCAGTGGCTAAGCGAAAGTGATAAGTATCCCACCTGGGGAGTACGTTCGCAAGAATGAA |
| cloaci08_sew_16S | ACGGAGGGTGCAAGCGTTATCCGGATTTATTGGGTTTAAAGGGTCCGTAGGCGGACTTATAAGTCAGTGGTGAAAGCCTGTCGCTTAACGATAGAACTGCCATTGATACTGTAAGTCTTGAGTATATTTGAGGTAGCTGGAATAAGTAGTGTAGCGGTGAAATGCATAGATATTACTTAGAACACCAATTGCGAAGGCAGGTTACCAAGATATAACTGACGCTGAGGGACGAAAGCGTGGGGAGCGAACAGGATTAGATACCCTGGTAGTCCACGCCGTAAACGATGCTAACTCGTTTTTGGGCTTTAGGGTTCAGAGACCAAGCGAAAGTGATAAGTTAGCCACCTGGGGAGTACGCTCGCAAGAGTGAA |
| cloaci32_sew_16S | ACGGAGGGTGCAAGCGTTATCCGGATTTATTGGGTTTAAAGGGTCCGTAGGCGGACTTATAAGTCAGTGGTGAAAGCCTGTCGCTTAACGATAGAACTGCCATTGATACTGTAAGTCTTGAGTATATTTGAGGTAGCTGGAATAAGTAGTGTAGCGGTGAAATGCATAGATATTACTTAGAACACCAATTGCGAAGGCAGGTTACCAAGATATAACTGACGCTGAGGGACGAAAGCGTGGGGAGCGAACAGGATTAGATACCCTGGTAGTCCACGCCGTAAACGATGCTAACTCGTTTTTGGAGCGCAAGCTTCAGAGACCAAGCGAAAGTGATAAGTTAGCCACCTGGGGAGTACGCTCGCAAGAGTGAA |

**Table S5.** Schema used to bin Human Microbiome Project (HMP) body site descriptions.

| **Binned body site name** | **HMP body site description** |
| --- | --- |
| Skin microbiome | L_Retroauricular crease |
|  | R_Retroauricular crease |
|  | L_Antecubital fossa |
|  | R_Antecubital fossa |
|  | Anterior nare |
| Oral | Saliva |
|  | Tongue dorsum |
|  | Hard palate |
|  | Buccal mucosa |
|  | Attached/Keratinized gingiva |
|  | Palatine Tonsils |
|  | Throat |
|  | Supragingival plaque |
|  | Subgingival plaque |
| Vaginal | Vaginal introitus |
|  | Mid vagina |
|  | Posterior fornix |
| Stool | Stool |

**
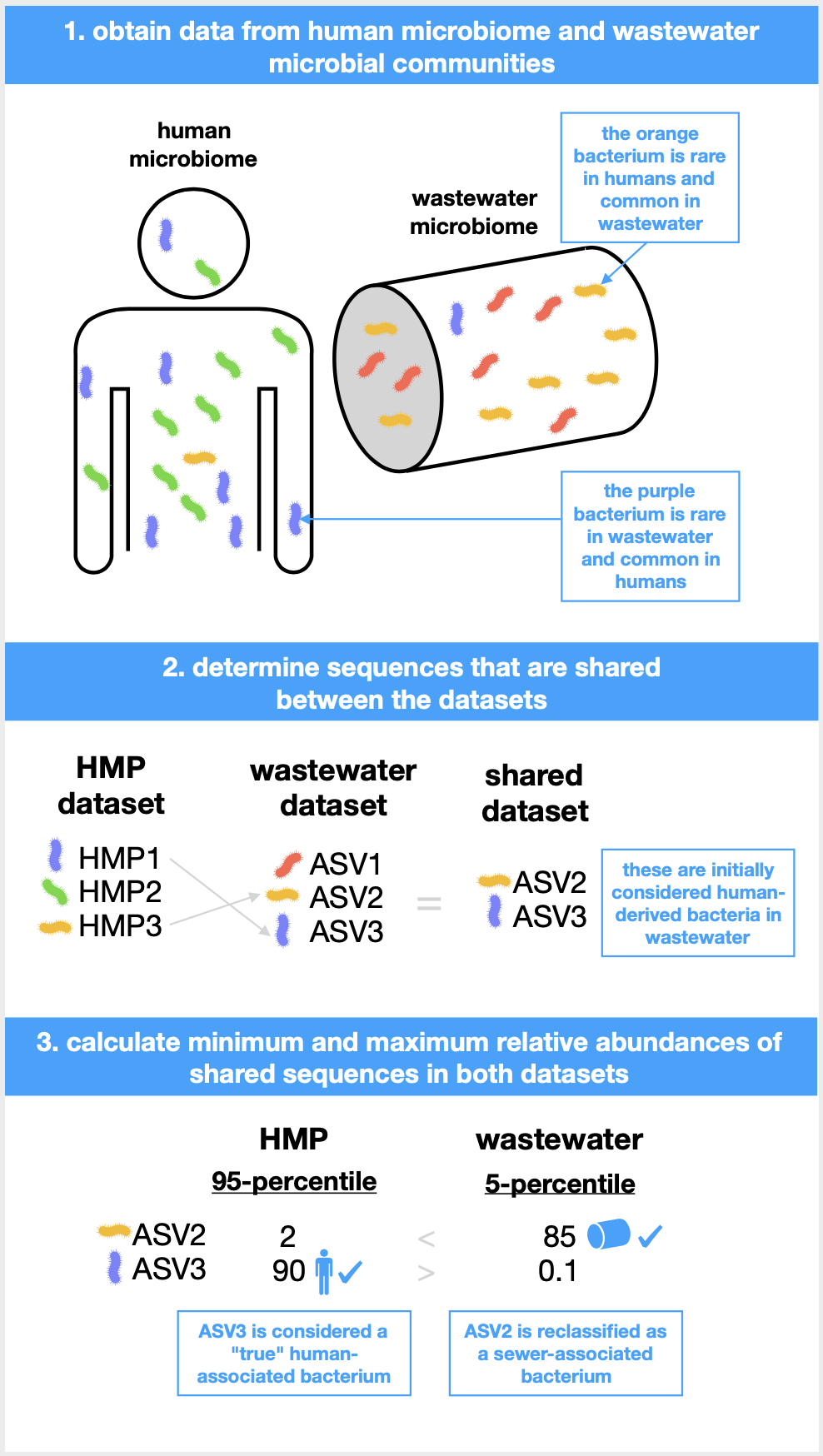
**

**Figure S3.** Diagram showing logic of microbiome classification threshold.

**Table S6.** R packages & datasets used for analysis.^1^

| **R package** | **Analysis** | **Function** | **Call** | **Purpose** |
| --- | --- | --- | --- | --- |
| dada2 (1.12.1) | Quality control and merge forward and reverse 16S rRNA gene amplicon sequences in FASTQ files (Table S4) | | | |
| phyloseq (1.28.0) | Organize ASV abundance matrices, taxonomy classifications, and sample information | | | |
| ggplot2 (3.2.1) | Create figures | | | |
| decontam (1.4.0) | Contaminant identification | *isContaminant* | isContaminant(phyloseq.object, method = "prevalence", neg = "NTC")  isContaminant(phyloseq.object, method = "prevalence", neg = "mock") | Identify and remove reads from the no template control and mock community |
| vegan (2.5.6) | Shannon alpha diversity | *diversity* | diversity(abundance.matrix, method = “shannon”) | Calculate alpha diversity within microbial community samples |
|  | Bray-Curtis dissimilarity | *vegdist* | vegdist(abundance.matrix, method = “bray”) | Calculate beta diversity between microbial community samples |
|  | Euclidian dissimilarity | *vegdist* | diversity(abundance.matrix, method = “euclidian”) | Calculate dissimilarity between normalized (z scores) ASV abundances |
|  | Constrained correspondence analysis (CCA) | *cca* | cca(abundance.matrix ~ ., sample.info, na.action = na.exclude) | Ordinate microbial communities against environmental variables |
|  | Environmental fit | *envfit* | envfit(cca.result ~ ., sample.info, perm = 999, na.rm = TRUE) | Fit environmental variables to CCA |
|  | PERMANOVA | *adonis* | adonis(abundance.matrix~season, data=season, permutations=100) | Test for statistical significance of month-based (season) bacterial community groupings |
| stats (3.6.0) | Shapiro-Wilk | *shapiro.test* | shapiro.test(vector) | Test for normal distributions |
|  | Mann-Whitney U | *wilcox.test* | wilcox.test(x = x.vector, y = y.vector, paired = FALSE, alternative = “greater”) | Compare alpha diversity measurements and cluster heights between datasets |
|  | Average hierarchical cluster | *hclust* | hclust(vegdist.object, method = “average”) | Cluster dissimilarity matrices to create dendrogram of ASVs based on abundance patterns |
|  | Centroid hierarchical cluster | *hclust* | hclust(vegdist.object, method = “centroid”) | Cluster dissimilarity matrices based on distance from center of clusters |
|  | Autocorrelation | *acf* | acf(vector, lag.max = 60, plot = FALSE)  # extract for ggplot  with(acf.result, data.frame(lag, acf)) | Observe seasonal abundance patterns of ASVs |
|  | Analysis of variance (ANOVA) | *aov* | aov(vector ~ variables) | Test if environmental metadata and ASV abundances are explained by season |
|  | Spearman rank correlation | *cor.test* | cor.test(vector.x, vector.y, method = “spearman”) | Correlate ASV abundances measured by 16S rRNA sequencing and ddPCR |
|  | Model prediction | *predict* | predict(aov.result, interval = “confidence”) | Make predictions from ANOVA |
| OTUtable (1.1.2) | Normalization | *zscore* | zscore(abundance.matrix) | Normalize ASV abundances to z scores ((x-μ)/σ) |
| indicspecies (1.7.6) | Multi-level pattern analysis (indicator species) | *multipatt* | multipatt(abundance.matrix, variables, min.order = 3, max.order = 3, control = how(nperm = 999) | Find associations between ASV abundances and groups of months (seasons) |
| ape (5.3) | Principal coordinate analysis (PCoA) | *pcoa* | pcoa(vegdist.object) | Visualize dissimilarities (as Bray-Curtis distances) between samples |

^1^For code, please visit our Github: https://github.com/NewtonLabUWM/Sewage_TimeSeries.

**Datasets**

- sample = 16S rRNA gene microbial community data generated using illumina sequencing of v4 variable region
- all samples relativized to relative abundance of ASVs (each ASV count is divided by the total sum; proportions)
- data partitioned into subgroups and/or transformed to z-score ((x-μ)/σ) will be stated accordingly

Shannon alpha diversity (R package *vegan*)

- calculated in all wastewater samples from all 3 datasets

Bray-Curtis dissimilarity (R package *vegan*)

- compare between JI samples
- compare between JI and SS samples
- compare between neighborhood samples
- compare between city samples
- compare between JI samples binned by month
- compare between JI samples binned by year
- between JI and SS samples in a PCoA
- between US samples binned by annual atmospheric temperature in PCoA
- compare between US samples binned by annual atmospheric temperature in PCoA and MKE WWTP samples binned by season in centroid cluster analysis
- compare among warm US cities, cold US cities, warm MKE WWTP season, cold WWTP season

Euclidian dissimilarity (R package *vegan*)

- between JI samples classified as "human-associated" normalized to z-score
- between JI samples classified as "sewer-associated" normalized to z-score
- between JI samples classified as "human-associated" normalized to z-score
- between JI samples classified as "sewer-associated" normalized to z-score

Principal coordinate analysis (R package *ape*)

- on Bray-Curtis dissimilarities between JI and SS WWTP samples

Mann-Whitney U (R package *stats*)

- test difference between beta diversity estimates in neighborhood and MKE WWTP samples (JI + SS)
- test if beta diversity in MKE WWTP samples is greater than MKE neighborhood samples
- test if hierarchical clustering data of Bray-Curtis distances between MKE WWTP samples is greater than those from city samples binned as “southern/warm”
- test if hierarchical clustering data of Bray-Curtis distances between city samples binned as “northern/cold” is greater than those from city samples binned as “southern/warm”

Multi-level pattern analysis (indicator species; R package *vegan*)

- within JI samples classified as "human-associated"
- within JI samples classified as "sewer-associated"

Constrained correspondent analysis (R package *vegan*)

- ordinate JI samples to JI metadata
- ordinate SS samples to SS metadata

ANOVA (R package *stats*)

- predict degree of variability in JI samples explained by JI metadata
- predict degree of variability in SS samples explained by SS metadata

Hierarchical clustering, “average” method (R package *stats*)

- on Euclidian distances between JI samples classified as “sewer-associated” for dendrogram
- on Euclidian distances between JI samples classified as “human-associated” for dendrogram

Hierarchical clustering, “centroid” method (R package *stats*)

- on Bray-Curtis distances between city samples binned by annual atmospheric temperature, JI samples, and SS samples

Temporal autocorrelation (R package *stats*)

- on *Cloacibacterium* indicator ASV in JI samples
- on *Flavobacterium* indicator ASV in JI samples
- on *Bacteroides* indicator ASV in JI samples

Spearman correlation

- between *Cloacibacterium* indicator ASV 16S rRNA gene sequencing data in JI samples and ddPCR data in JI samples
- between *Flavobacterium* indicator ASV 16S rRNA gene sequencing data in JI samples and ddPCR data in JI samples
- between *Bacteroides* indicator ASV 16S rRNA gene sequencing data in JI samples and ddPCR data in JI samples
- between *Cloacibacterium* indicator ASV 16S rRNA gene sequencing data in JI samples and water temperature of JI samples
- between *Flavobacterium* indicator ASV 16S rRNA gene sequencing data in JI samples and water temperature of JI samples
- between *Bacteroides* indicator ASV 16S rRNA gene sequencing data in JI samples and water temperature of JI samples

Shapiro-Wilk

- test for normal distributions in hierarchical clustering data of Bray-Curtis distances between city samples binned by annual atmospheric temperature, JI samples, and SS samples

Environmental fit

- on results of CCA of JI samples to JI metadata
- on results of CCA of SS samples to SS metadata

**Table S7.** Indicator species (ASVs) in three-month windows of the Jones Island time series.

| **Genus** | **ASV** | **p value** | **Window** |
| --- | --- | --- | --- |
| *Arcobacter* | ASV124 | 0.001 | FebMarApr |
| *Flavobacterium* | ASV42 | 0.002 | FebMarApr |
| *Polaromonas* | ASV103 | 0.001 | MarAprMay |
| *Pseudomonas* | ASV214 | 0.001 | MarAprMay |
| *Rhodoferax* | ASV69 | 0.001 | MarAprMay |
| *Arcobacter* | ASV13 | 0.001 | MarAprMay |
| *Acinetobacter* | ASV94 | 0.024 | MarAprMay |
| *Simplicispira* | ASV41 | 0.007 | AprMayJun |
| *Flavobacterium* | ASV612 | 0.001 | AprMayJun |
| *Arcobacter* | ASV4 | 0.001 | AugSepOct |
| *Enterobacter* | ASV48 | 0.001 | AugSepOct |
| *Macellibacteroides* | ASV14 | 0.001 | SepOctNov |
| *Cloacibacterium* | ASV8 | 0.001 | SepOctNov |
| *Sphaerotilus* | ASV88 | 0.005 | OctNovDec |

**Table S8.** Environmental fit scores (R^2^ and p values) of microbial communities at Jones Island and South Shore influent fit to environmental metadata.

| **Variables** | **South Shore** | | **Jones Island** | |
| --- | --- | --- | --- | --- |
|  | **R^2^** | **p value** | **R^2^** | **p value** |
| Month | 0.6346 | 0.033 | 0.5626 | 0.001 |
| Year | 0.3483 | 0.010 | 0.1178 | 0.028 |
| Wastewater temperature (°F) | 0.9724 | 0.001 | 0.9579 | 0.001 |
| Air temperature (°F) | 0.4870 | 0.001 | 0.2439 | 0.001 |
| Precipitation (48 hours) | 0.3644 | 0.013 | 0.0035 | 0.895 |
| Flow (millions/gal/day) | 0.8534 | 0.001 | 0.1576 | 0.041 |
| Ammonia (mg/L) | 0.5800 | 0.001 | 0.2717 | 0.001 |
| BOD5 (mg/L) | 0.3804 | 0.010 | 0.2798 | 0.001 |
| Phosphorus (mg/L) | 0.4515 | 0.002 | 0.1678 | 0.027 |
| TSS (mg/L) | 0.5106 | 0.002 | 0.0537 | 0.164 |

**
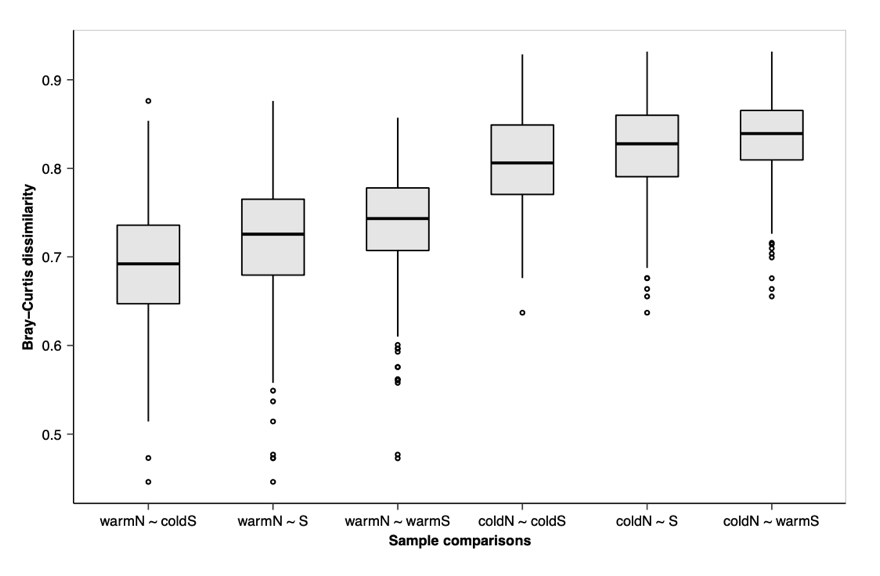
**

**Figure S4.** Bray-Curtis dissimilarity measurements comparing samples between Northern US and Milwaukee time series in the January-May sampling period (coldN), Northern US and Milwaukee time series in the August sampling period (warmN), Southern US during the January-May sampling period (coldS), Southern US during the August sampling period (warmS), and Southern US from all sampling periods (S).


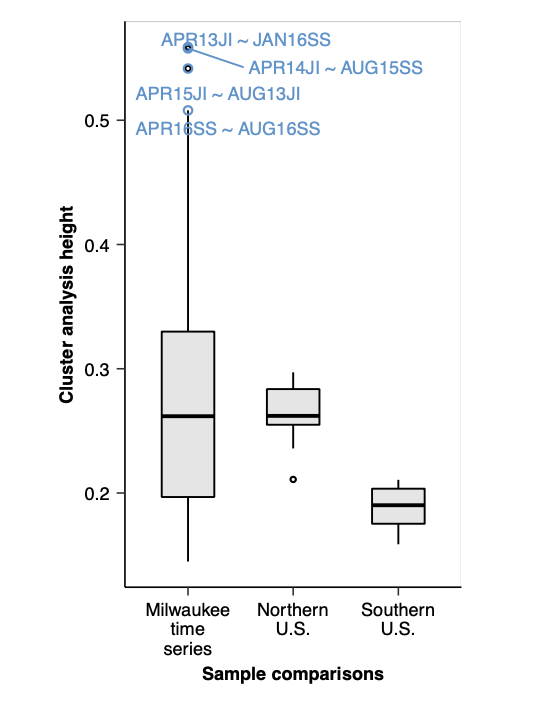


**Figure S5.** Centroid cluster analysis community composition distances of the ten warmest WWTPs (Southern US), ten coolest WWTPs (Northern US), and the Milwaukee time series. Blue points are outliers (height > 0.5). Point labels indicate the month, year, and WWTP (JI or SS) of the sample.
